# The complete mitochondrial genome of *Sympiezomias velatus* (Coleoptera: Curculionidae)

**DOI:** 10.1080/23802359.2017.1357445

**Published:** 2017-07-25

**Authors:** Pei-An Tang, Li Zhang, Xiao-Peng Li, Fei-Fan Li, Ming-Long Yuan

**Affiliations:** a Collaborative Innovation Center for Modern Grain Circulation and Safety, College of Food Science and Engineering, Nanjing University of Finance and Economics, Nanjing, China;; b State Key Laboratory of Grassland Agro-Ecosystems, College of Pastoral Agricultural Science and Technology, Lanzhou University, Lanzhou, China

**Keywords:** Beetles, weevils, Entiminae, mitochondrial genomes, phylogeny

## Abstract

Here, we determined the complete mitogenome sequence of *Sympiezomias velatus* (Coleoptera: Curculionidae: Entiminae). This mitogenome was 15,592 bp long with an A + T content of 74.1% and contains 13 protein-coding genes (PCGs), 21 transfer RNA genes (tRNAs), 2 ribosomal RNA unit genes and a large non-coding region (putative control region). The *trnI* gene was not found in the *S. velatus* mitogenome. The order and orientation of the mitochondrial genes were identical to the inferred ancestral arrangement of insects except for *trnR* which was changed to be adjacent the *nad3* gene. All tRNAs had the typical cloverleaf structure, except for *trnS1* which lacked the dihydrouridine arm. The Bayesian phylogenetic tree of 10 Entiminae species based on the concatenated nucleotide sequences of 13 PCGs strong supported a sister relationship of *S. velatus* and *Barynotus obscures*.

The genus *Sympiezomias* belongs to the Entiminae, a large subfamily in the weevil family Curculionidae. *Sympiezomias velatus* is an important polyphagous pest on many crops and fruit trees in China. Here, we sequenced and annotated the complete mitogenome of *S. velatus*, following the methods described in (Yuan et al. 2016). Adult specimens were collected from Xifeng District, Qingyang City, Gansu Province, China, in July 2014. Samples have been deposited in College of Pastoral Agricultural Science and Technology, Lanzhou University, Lanzhou, China.

The complete mitogenome of *S. velatus* was a typical circular DNA molecule with 15,592 bp in length (GenBank accession no. MF383367). This mitogenome contained 13 protein-coding genes (PCGs), 21 transfer RNA genes (tRNAs), the large and small ribosomal RNA unit genes (*rrnL* and *rrnS*), and a large non-coding region (putative control region). The *trnI* was not detected in the *S. velatus* mitogenome, as observed in *Naupactus xanthographus* (Song et al. 2010), another completely sequenced species within Entiminae. The order and orientation of the mitochondrial genes are identical to the inferred ancestral arrangement of insects (Boore 1999), except for a tRNA rearrangement. Typically, the ancestral order of six tRNAs between *nad3* and *nad5* is ARNSEF, whereas *S. velatus* exhibited RANSEF, as reported in all the species of Entiminae (Song et al. 2010; Haran et al. 2013). Two large gene overlaps, i.e. *atp8*/*atp6* (–7 bp) and *nad4*/*nad4L* (–7 bp), were present in the *S. velatus* mitogenome, whereas a total of 45 bp intergenic spacers were present in 11 positions, ranging in size from 1 to 17 bp.

The mitogenome of *S. velatus* with an A + T content of 74.1% presented a positive AT-skew (0.037) and a negative GC-skew (–0.250) on the J-strand, as reported in most insect mitogenomes. Eleven PCGs started with a typical ATN codon: one (*cob*) with ATA, two (*atp8* and *nad5*) with ATT, four (*atp6*, *cox3*, *nad4*, and *nad4L*) with ATG and four (*nad2*, *cox2*, *nad3*, and *nad6*) with ATC. The remaining two PCGs started with TTG (*nad1*) or TAT (*cox1*). Five PCGs terminated with TAA, one terminated with TA (*atp6*), whereas the remaining seven terminated with an incomplete stop codon T. All of the 21 tRNAs, ranging from 63 bp (*trnC* and *trnE*) to 76 bp (*trnG*), had a typical cloverleaf structure, except for *trnS1* which lacked the dihydrouridine arm. The *rrnL* was 1293 bp long with an A + T content of 78.5%, and the *rrnS* was 786 bp with an A + T content of 74.6%.

The concatenated nucleotide sequences of 13 PCGs from 10 Entiminae species and outgroups from the subfamily Hyperinae (*Hypera plantaginis*) were used for the phylogenetic analysis. The optimal partitioning schemes and corresponding nucleotide substitution models were determined by PartitionFinder v1.1.1 (Lanfear et al. 2012). Bayesian analyses were performed with MrBayes 3.2.6 (Ronquist et al. 2012). The results showed that *S. velatus* clustered with *Barynotus obscures* with a high support value (Figure 1). This is the second completely sequenced mitogenome from the subfamily Entiminae of Curculionidae.

**Figure 1.**
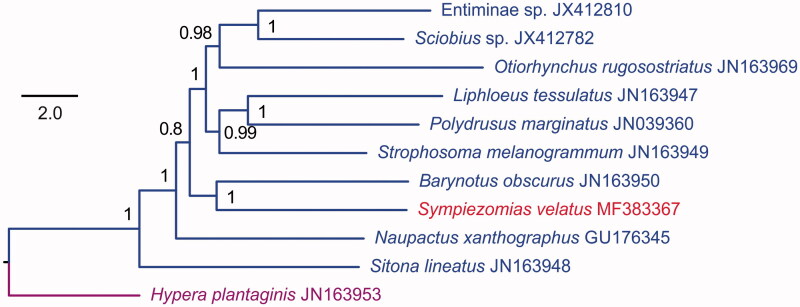
Mitochondrial phylogeny of 10 Entiminae species based on the concatenated nucleotide sequences of 13 mitochondrial protein-coding genes.
